# The influence of network structure on neuronal dynamics

**DOI:** 10.1186/1471-2202-14-S1-P45

**Published:** 2013-07-08

**Authors:** Patrick Campbell, Duane Q Nykamp, Michael Buice

**Affiliations:** 1 School of Mathematics, University of Minnesota, Mineapolis, MN 55455, USA; 2 Allen Institute for Brain Science, Seattle, WA 98103, USA

## 

**Figure 1:**
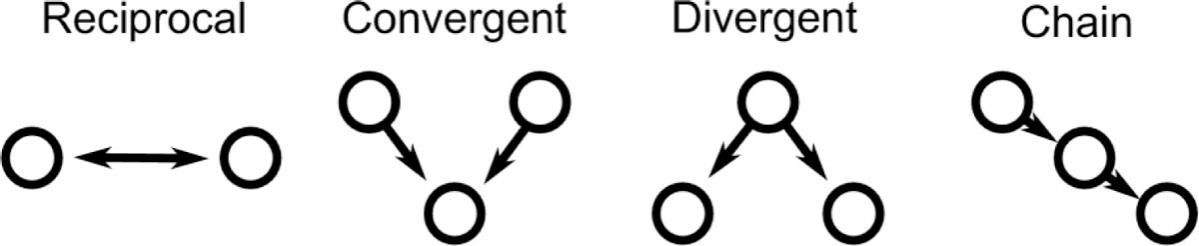
**The four second order edge motifs of reciprocal, convergent, divergent, and causal connections**. The frequency of these motifs determine the second order statistics, or correlations, among the network connections.

Understanding the influence of network structure on neural dynamics is a fundamental step toward deciphering brain function, yet presents many challenges. We show how networks may be described in terms of the occurrences of certain patterns of edges, and how the frequency of these *motifs *(see Figure 1) impacts global dynamics. Through analysis and simulation of neuronal networks, we have found that two edge directed paths (*two-chains*) have the most dramatic effect on dynamics. Our analytic results are based on equations for mean population activity and correlations that we derive using path integrals and moment hierarchy expansions. These equations reveal the primary ways in which the network motifs influence dynamics. For example, the equations indicate that the propensity of a network to globally synchronize increases with the prevalence of two-chains, and we verify this result with network simulations. Finally, we present ongoing work investigating when these second-order equations break down, and how they might be corrected by higher order approximations to the network structure, such as the prevalence of three edge chains beyond that predicted by the two-chains.

